# Comparison of pharmacological and non-pharmacological interventions to prevent delirium in critically ill patients: a protocol for a systematic review incorporating network meta-analyses

**DOI:** 10.1186/s13643-016-0327-0

**Published:** 2016-09-08

**Authors:** L.D. Burry, B. Hutton, M. Guenette, D. Williamson, S. Mehta, I. Egerod, S. Kanji, N.K. Adhikari, D. Moher, C.M. Martin, L. Rose

**Affiliations:** 1 Leslie Dan Faculty of Pharmacy, University of Toronto, Toronto, ON Canada; 2 Department of Pharmacy, Mount Sinai Hospital, 600 University Ave, Rm 18-377, Toronto, ON M5G1X5 Canada; 3 Clinical Epidemiology Program, Ottawa Hospital Research Institute, The Ottawa Hospital – General Campus, 501 Smyth Rd, Room L1288, Ottawa, ON K1H8L6 Canada; 4 School of Epidemiology, Public Health and Preventive Medicine, University of Ottawa, Ottawa, ON Canada; 5 Faculté de pharmacie, Université de Montréal, Montreal, QC Canada; 6 Département de pharmacie, hôpital du Sacré-Coeur, Montreal, QC Canada; 7 Department of Medicine, Interdepartmental Division of Critical Care, University of Toronto, Toronto, ON Canada; 8 Department of Medicine, Division of Critical Care, Mount Sinai Hospital, Toronto, ON Canada; 9 University of Copenhagen, Rigshospitalet, Neurointensive Intensive Care, Blegdamsvej 9, DK-2100 Copenhagen O, Denmark; 10 Department of Pharmacy, The Ottawa Hospital, Ottawa, ON Canada; 11 Department of Critical Care Medicine, Sunnybrook Health Sciences Centre, Toronto, ON Canada; 12 Evaluative Clinical Sciences, Trauma, Emergency & Critical Care Research Program, Sunnybrook Research Institute, Toronto, ON Canada; 13 Department of Medicine, Division of Critical Care, Schulich School of Medicine and Dentistry, Western University, London, ON Canada; 14 Lawson Health Research Institute, London Health Sciences Centre, London, ON Canada; 15 Lawrence S. Bloomberg Faculty of Nursing, University of Toronto, Toronto, ON Canada; 16 Institute for Clinical Evaluative Sciences, Toronto, ON Canada; 17 Provincial Centre of Weaning Excellence, Toronto East General Hospital, Toronto, ON Canada

**Keywords:** Delirium, Prevention, Intensive care unit, Network meta-analysis

## Abstract

**Background:**

Delirium is characterized by acute changes in mental status including inattention, disorganized thinking, and altered level of consciousness, and is highly prevalent in critically ill adults. Delirium has adverse consequences for both patients and the healthcare system; however, at this time, no effective treatment exists. The identification of effective prevention strategies is therefore a clinical and research imperative. An important limitation of previous reviews of delirium prevention is that interventions were considered in isolation and only direct evidence was used. Our systematic review will synthesize all existing data using network meta-analysis, a powerful statistical approach that enables synthesis of both direct and indirect evidence.

**Methods:**

We will search Ovid MEDLINE, CINAHL, Embase, PsycINFO, and Web of Science from 1980 to March 2016. We will search the PROSPERO registry for protocols and the Cochrane Library for published systematic reviews. We will examine reference lists of pertinent reviews and search gr﻿ey literature and the International Clinical Trials Registry Platform for unpublished studies and ongoing trials. We will include randomized and quasi-randomized trials of critically ill adults evaluating any pharmacological, non-pharmacological, or multi-component intervention for delirium prevention, administered in or prior to (i.e., peri-operatively) transfer to the ICU. Two authors will independently screen search results and extract data from eligible studies. Risk of bias assessments will be completed on all included studies. To inform our network meta-analysis, we will first conduct conventional pair-wise meta-analyses for primary and secondary outcomes using random-effects models. We will generate our network meta-analysis using a Bayesian framework, assuming a common heterogeneity parameter across all comparisons, and accounting for correlations in multi-arm studies. We will perform analyses using WinBUGS software.

**Discussion:**

This systematic review will address the existing knowledge gap regarding best practices for delirium prevention in critically ill adults by synthesizing evidence from trials of pharmacological, non-pharmacological, and multi-component interventions administered in or prior to transfer to the ICU. Use of network meta-analysis will clarify which delirium prevention strategies are most effective in improving clinical outcomes while causing least harm. The network meta-analysis is a novel approach and will provide knowledge users and decision makers with comparisons of multiple interventions of delirium prevention strategies.

**Systematic review registration:**

PROSPERO CRD42016036313

**Electronic supplementary material:**

The online version of this article (doi:10.1186/s13643-016-0327-0) contains supplementary material, which is available to authorized users.

## Background

Delirium is a syndrome commonly experienced by patients in the intensive care unit (ICU), and is characterized by acute changes in mental status including inattention, disorganized thinking, and altered level of consciousness [1]. Reported delirium prevalence rates range from 60 to 89 % in mechanically ventilated, and 40 to 60 % in non-ventilated, critically ill patients [2, 3]. Numerous studies have explored risk factors associated with the development of delirium in critically ill populations, with varying results. In a recent systematic review of 33 studies, significant risk factors included increased age, dementia, hypertension, ICU admission due to emergency surgery or trauma, higher illness severity [4], mechanical ventilation, metabolic acidosis, history of delirium, and coma [5]. Delirium in critically ill patients has been associated with multiple adverse patient consequences such as prolonged duration of mechanical ventilation and length of ICU and hospital stay, as well as increased mortality and greater likelihood of long-term cognitive impairment, functional decline, and placement in long-term care facilities [6–13].

At this time, there are no effective treatments for ICU delirium; [14, 15] it is therefore imperative to explore potential *prevention* strategies. Such strategies fall broadly into pharmacological, non-pharmacological, and multi-component interventions, and can be administered once in the ICU or prior to (e.g., peri-operative) admission. Pharmacological interventions include antipsychotics (e.g., haloperidol), sedatives (e.g., benzodiazepines, propofol), alpha-agonists (e.g., dexmedetomidine, clonidine), cholinesterase inhibitors (e.g., rivastigmine), melatonin and melatonin receptor agonists, HMG-CoA reductase inhibitors (statins), and anesthetics. Non-pharmacological strategies include interventions to promote sleep (e.g., noise and light reduction), relaxation (e.g., touch, music), movement (e.g., early mobilization), and patient orientation [14, 16]. Non-pharmacological strategies may be evaluated singularly, but are more often employed as multi-component approaches designed to address delirium risk factors such as cognitive impairment, sleep deprivation, immobility, visual and hearing impairment, and dehydration.

The Society of Critical Care Medicine’s 2013 Pain, Agitation, and Delirium (PAD) guidelines state that there is no compelling evidence to make specific recommendations for any pharmacological intervention to reduce delirium incidence [14]. A recent (2015) systematic review [17] of pharmacological strategies for the prevention and treatment of ICU delirium, however, reported that while pharmacological interventions were not associated with a significant reduction in delirium prevalence or duration of mechanical ventilation, these did show a possible favorable effect on length of ICU stay. As it pertains to trials evaluating non-pharmacological or multi-component interventions, these have shown positive results in hospitalized, non-critically ill patients [18, 19], and numerous trials are underway in critically ill populations and will be available for consideration in the near future. The Society of Critical Care Medicine’s 2013 PAD guidelines recommend the non-pharmacological strategy of early mobilization whenever possible to reduce the incidence and duration of delirium.

An important limitation of previous systematic reviews on delirium prevention is that interventions were considered in isolation and only direct evidence from head-to-head comparisons was used. Our systematic review will synthesize existing data from identified trials using network meta-analysis (NMA), a powerful statistical approach that enables synthesis of both direct and indirect evidence in a multi-treatment analytical framework [20–22]. This approach will allow the assessment of the relative efficacy and safety of interventions that may or may not have been directly compared in randomized controlled trials. We will also expand the scope of considered interventions to include anesthetic drug manipulations made prior to transfer to the ICU (e.g., intra-operatively) and sedation administration strategies (e.g., daily sedation interruption or protocolized sedation) employed in the ICU.

The primary objective of this systematic review is to compare interventions for delirium prevention (pharmacological and non-pharmacological) in critically ill adults using NMA, thus informing clinicians and other knowledge users of the safest and most effective strategies. The assessment of the comparative benefits and harms of each intervention via NMA will permit the ranking of interventions according to their effectiveness and acceptability, therefore informing policy and clinical decision-making.

## Methods

This systematic review protocol was prepared using the Preferred Reporting Items for Systematic Reviews and Meta-Analyses Protocol (PRISMA-P) guidelines [23]. A PRISMA-P checklist was completed (Additional file 1). The protocol for this review has been registered on the PROSPERO International Prospective Register of Systematic Reviews (CRD42016036313).

### Data sources and search strategy

We created a preliminary search strategy (Additional file 2) with the assistance of an experienced senior information specialist. A second senior information specialist reviewed the search strategy prior to its execution using the Peer Review for Electronic Search Strategies (PRESS) template [24, 25]. We will search the following electronic databases from 1980 to March 2016: Ovid MEDLINE, Ovid MEDLINE In-Process & Other Non-Indexed Citations, CINAHL, Embase Classic + Embase, PsycINFO, and Web of Science. Search terms will include extensive controlled vocabulary and keywords such as “intensive care unit” and “delirium.” We will use a validated randomized controlled trial filter and perform a separate search for published systematic reviews in the Cochrane Library and in PROSPERO. We will review reference lists of relevant trials and reviews for additional studies not identified through our electronic search methods. We will perform a grey literature search using sources listed in the Canadian Agency for Drugs and Technologies in Health’s (CADTH) Grey Matters [26]. We will search for unpublished studies and ongoing trials on the International Clinical Trials Registry Platform (http://apps.who.int/trialsearch).

### Study eligibility criteria

We will include randomized controlled trials, including those using open-label and quasi-randomized (i.e., quasi-random method of allocation such as alternation) designs. We will include studies that evaluate any intervention designed to prevent delirium in critically ill adults. We will exclude studies with non-randomized designs (e.g., cohort studies, case reports, case series).

#### Population

Our population of interest is critically ill adults aged 16 years and older, and treated in an ICU or critical care unit of any type (e.g., medical, surgical, trauma, mixed, burn, cardiac, or cardiac surgery) or in a high-acuity unit. For trials enrolling both ICU and non-ICU patients, we will include the following: trials where a minimum of 50 % of subjects are ICU patients, or those where fewer than 50 % of subjects are ICU patients but that report outcome data for the ICU group distinct from that of the total study population. We will also include trials where ﻿an intervention is applied outside of the ICU (e.g., in the operating room), so long as the patient population is expected to be trans﻿ferred to the ICU (e.g., cardiac surgery). Trials including patients confirmed as delirious at enrolment will be excluded. However, we will include trials where some or all patients are deemed to have sub-syndromal delirium [27, 28].

#### Interventions

We will include trials that evaluate any intervention designed to prevent delirium in critically ill adults, or that evaluate interventions not specif﻿ically targeted to delirium prevention but ﻿necessarily include incidence as an a priori-defined outcome that is measured across treatment groups (e.g., sedation administration strategies). Interventions may comprise, but are not limited to, any pharmacological (e.g., typical and atypical antipsychotics, benzodiazepines, alpha-2 agonists, opioids, cholinesterase inhibitors, melatonin, melatonin receptor agonists, propofol), non-pharmacological (e.g., noise reduction, early mobilization, music therapy, acupuncture), or multi-component strategy. Additionally, we will include trials that evaluate sedative drugs (e.g., propofol versus midazolam) and/or sedation administration strategies (e.g., protocolized sedation, daily sedation interruption, intermittent dosing, continuous dosing, opioid only sedation) that report delirium incidence as a primary or secondary outcome. Such studies will be included because sedative exposure has been identified as a potentially modifiable risk factor for delirium [5, 14, 29–32]. Additionally, sedation administration strategies in critically ill patients generally aim to achieve sufficient wakefulness, enabling patients to be assessed for delirium and participate in reorientation and early mobilization strategies [29]. Lastly, we will include studies in which pre- and intra-operative interventions are applied in patients admitted post-operatively to the ICU (e.g., cardiac surgery) that report delirium incidence as a primary or secondary outcome. Such studies can include those in which interventions for delirium prevention are investigated specifically (e.g., dexmedetomidine), or those in which routinely applied drugs (e.g., analgesic and anesthetic agents) and/or intra-operative techniques (e.g., depth of anesthesia) are compared.

#### Comparators

**Fig. 1:**
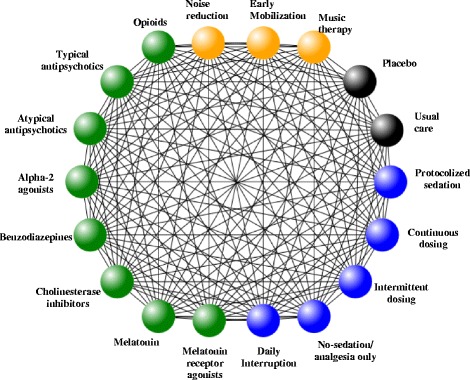
Interventions eligible for network meta-analysis. *Lines* reflect where comparisons may exist between treatments. Which comparisons have been studied will be established by studies identified. Availability of outcomes can also impact network structure. Clinical experts have guided the network refinement

An NMA is capable of estimating comparisons of multiple interventions based on both direct and indirect evidence [20–22]. Using this unique approach, we will include all trials evaluating any of the aforementioned strategies, regardless of the intervention used in the control arm (i.e., whether the study compares one delirium prevention strategy to another, placebo, or care as usual (Fig. 1)). Descriptions of care as usual will be extracted verbatim from studies to ensure that similarities and differences are appropriately reflected in the NMA.

#### Outcomes

Our primary outcome of interest is delirium incidence, defined as at least one episode of delirium experienced during ICU admission and identified using either a validated delirium screening tool (e.g., Confusion Assessment Method (CAM-ICU) [33], Intensive Care Delirium Screening Checklist (ICDSC) [34], or the Neelon and Champagne (NEECHAM) Confusion Scale [35]), th﻿e assessment of a trained individual (e.g., psychiatrist) using Diagnostic and Statistical Manual of Mental Disorders IV or V criteria [1, 36], or as otherwise defined by study authors.

Secondary outcomes include the following: number of delirium- and coma-free days, total duration of delirium, delirium severity, incidence of sub-syndromal delirium (for those without sub-syndromal detected on enrolment) [27, 28], defined as the presence of sub-threshold delirium symptoms that do not progress to delirium, duration of mechanical ventilation, duration of ICU and hospital length of stay, mortality, adverse events (e.g., QT interval prolongation, seizures), use of physical restraints, disposition at hospital discharge, and long-term cognitive and health-related quality of life outcomes after hospital discharge. As mortality may be defined at various time points, we will extract all reported mortality outcomes and create subgroups where needed for descriptive and analytical purposes.

### Screening and data extraction

Two authors (LB, MG) will independently screen search results against eligibility criteria to identify potentially relevant studies. We will perform a calibration exercise prior to screening to ensure inter-rater reliability by pilot testing the predesigned screening form on a sample of five studies. References will be organized using the reference management software package EndNote (X7 edition, Thomson Reuters, available at http://endnote.com/). The full text version of publish﻿e﻿d m﻿an﻿uscri﻿pts, o﻿r abstracts﻿, in the case of unpublished or ongoing studies, identified by either author, will be examined independently to confirm inclusion; reasons for exclusion will be listed in the notes field of EndNote. Any disagreement will be discussed with an independent arbiter (LR). We will present a summary of the search and study selection process using a PRISMA flow diagram [37].

As we anticipate a large number of eligible trials, we will divide studies among pairs of authors (DW/SM, IE/SK, and NA/MG). Each member of an author pair will independently extract data using a standardized electronic data extraction form using Microsoft Excel version 14.6.2 (Microsoft Corporation, Seattle, WA, USA). We have piloted the extraction form on five studies to ensure it captures all relevant data. We will extract data on participant demographics (e.g., age, gender, severity of illness score, reasons for ICU admission), verbatim descriptions of delirium prevention and comparator arm interventions, methods of delirium assessment, use of sedation and pain scales, and any co-interventions that may influence the development of delirium such as avoidance of medications that may be associated with delirium (e.g., benzodiazepines), as well as data on our selected outcomes of interest.

We will contact study corresponding authors as necessary to clarify issues related to data reporting, risk of bias, or to obtain further study details. All data extraction will be confirmed (LB, MG) and, where necessary, any discrepancy will be resolved by an independent arbiter (LR).

### Study risk of bias assessment

Each data extractor will independently assess risk of bias of their assigned studies, and a third author (LR) will confirm the final bias assessment. We will use a﻿ domain-based evaluation for risk of bias assessment, as recommended by the Cochrane Collaboration [38]. The domains are as follows: (1) random sequence generation (i.e., selection bias); (2) allocation concealment (i.e., selection bias); (3) blinding of participants and personnel (i.e., performance bias); (4) blinding of outcomes assessment (i.e., detection bias); (5) incomplete outcome data (i.e., attrition bias); (6) selective reporting; and (7) other bias (e.g., source of funding). For hard endpoints such as mortality, incidence of delirium, and duration of mechanical ventilation, a lack of blinding does not immediately warrant a judgment of high risk of bias as this aspect of study design is not considered to have a biasing effect on the endpoints. Each endpoint and the risk of bias will be assessed individually to generate an overall score.

For each domain, we will assess the risk of bias as “low,” “high,” or “unclear.” Unclear risk will be assigned for a domain if insufficient detail is reported and cannot be obtained from study authors, or if what happened in the study is known, but its contribution to the risk of bias is unknown or unclear. After ri﻿sk of bias assessment , studies will be classified according to the following categories:

1. Low risk: studies where all domains are considered to be at “low” risk of bias;
2. High risk: studies where one or more domains are considered to be at “high” risk of bias; and
3. Unclear risk: studies where one or more domain(s) have “unclear” risk of bias.

### Approach to evidence synthesis

In the NMA each intervention will have its own node (Fig. 1). If we encounter combined interventions of both pharmacological and non-pharmacological strategies, the research team will be consulted to determine the best approach for analysis.

To inform the NMA, we will first conduct conventional pair-wise meta-analyses for the primary and all secondary outcomes using random-effects models, provided at least two studies reporting a given outcome are available [39]. Pair-wise meta-analyses will be generated for each comparison of unique interventions within the final treatment network.

The research team will collectively review and discuss extracted characteristics of included studies to assess the extent of clinical and methodological diversity. We will summarize characteristics of included studies focusing on clinical (e.g., type of delirium prevention strategy) and methodological (e.g., risk of bias) homogeneity. We will review the distribution of these potential effect modifiers across studies in the network to determine the validity of the assumptions of homogeneity and similarity. These analyses, along with corresponding inspections of *I*^2^ values and distributions of treatment effects, will permit judgments about whether sufficient homogeneity exists within comparisons in the treatment network. If homogeneity is established, we will progress to performing the planned NMA.

We will generate the NMA using a Bayesian framework, assuming a common heterogeneity parameter across all comparisons and accounting for correlations in multi-arm studies [40, 41]. We will perform analyses using WinBUGS software [42] (version 1.4.3, MRC Biostatistics Unit, Cambridge, UK) (http://www.mrc-bsu.cam.ac.uk/software/bugs/the-bugs-project-winbugs/) through well-established methods.

We will express continuous and binary outcomes in terms of mean differences and odds ratios, respectively, with corresponding 95 % confidence intervals [43–45]. Secondary summary measures including treatment rankings and Surface Under the Cumulative Ranking (SUCRA) curve will also be provided [41]. We will evaluate adequacy of model fit through comparison of the posterior residual deviance with the number of unconstrained data points (i.e., the total number of interventions across studies) [45]. Adequate fit is deemed present when these quantities are approximately equal. Both fixed- and random-effects consistency models will be run, and their fit will be compared using the Deviance Information Criterion, which penalizes model fit for complexity (lower values indicate better models) [46]. A difference of approximately five points or more will be considered indicative of an important difference. Inconsistency models will also be fitted, and we will compare their Deviance Information Criterion values with those of consistency models to explore the presence of inconsistency. If inconsistency is detected, individual study characteristics will be examined, as will the need for additional statistical considerations such as meta-regression. We will assess model convergence using established methods including Gelman Rubin diagnostics and inspection of Monte Carlo errors [45].

### Subgroup and sensitivity analyses

We will explore subgroup analyses and/or meta-regression analyses to address the impact of covariates on our findings to establish their robustness. If we identify sufficient data, we will seek to determine if the efficacy and safety of prevention strategies are influenced by (1) age (<65, ≥65 years) and (2) ICU population (e.g., mixed, medical only, surgical only).

We will conduct a sensitivity analysis excluding studies at high risk of bias. Additionally, we will conduct a sensitivity analysis involving alternative geometries of the network. Examples of potential reformulation of the network include (1) collapsing atypical and typical antipsychotics into one node and (2) splitting each node in Fig. 1 into two to reflect “low” and “high” drug doses.

### Reporting of review findings

We will adhere to recommendations from the PRISMA-NMA extension statement on NMA for reporting our review findings [47]. We will include recommended graphical approaches such as forest plots, league tables, and rank-o-grams [41]. We will provide a summary of the geometries of the networks to provide insight for future clinical trials.

### Dissemination of findings

We have chosen to use the knowledge-to-action framework [48–50], which emphasizes the successful implementation of research evidence into practice in two phases: (1) knowledge creation and (2) action. We will solicit input from knowledge users and key stakeholder in order t﻿o generate project recommendations and knowledge translation opportunities. We will formally communicate our findings via summary documents, with versions tailored to specific audiences (e.g., patients and family members, ﻿clinicians, researchers, policy makers), presentations delivered at local, national, and international forums, and publication﻿s in peer-reviewed journals.﻿ We will hold an end-of-study workshop where key stakeholders will discuss findings, provide feedback on summary documents, and strategize further knowledge dissemination strategies. We will distribute summaries to various relevant advocacy programs.

## Discussion

This systematic review will address the existing knowledge gap regarding best practice for delirium prevention in critically ill adults. An NMA approach to evidence synthesis will be used to evaluate trials employing pharmacological, non-pharmacological, and multi-component interventions for delirium prevention, enabling the identification of strategies that most improve clinical outcomes while causing least harm. Use of NMA to evaluate delirium prevention interventions is novel and will provide knowledge users and decision-makers with comparisons of multiple interventions.

The NMA is an important but as of yet under-utilized method of evaluating interventions for the prevention of delirium in critically ill patients. In a field where a substantial body of evidence exists, clinicians may be unable to establish which approach is most effective because only traditional pair-wise comparisons are available to support guideline development and inform clinical practice. Given that multiple types of delirium prevention interventions are currently in use, we believe a new systematic review that employs a more sophisticated approach to evidence synthesis is warranted. The use of NMA will provide clinicians and researchers with clarity regarding the relative effectiveness and safety of available options, as well as enabling the identification of patients most likely to benefit. Further, we will provide researchers with a solid framework of current knowledge and identify evidence gaps to guide future research.

### Registration

This systematic review is registered with PROSPERO, an international prospective register of systematic reviews (http://www.crd.york.ac.uk/PROSPERO/display_record.asp?ID=CRD42016036313).

## Additional files

Additional file 1: Preferred Reporting Items for Systematic Review and Meta-Analyses Protocol (PRISMA-P). PRISMA-P items and their respective line locations within the manuscript. (DOCX 135 kb) Additional file 2: Preliminary search strategy. Preliminary search strategy, including all queried databases, search parameters, and key words. (DOCX 112 kb)

## Abbreviations

ICU: Intensive care unit
NMA: Network meta-analysis
PRISMA: Preferred Reporting Items for Systematic Reviews and Meta-Analyses
