# Supplementary material for: Comparison of pharmacological and non-pharmacological interventions to prevent delirium in critically ill patients: a protocol for a systematic review incorporating network meta-analyses
Source: Syst Rev. 2016 Sep 8;5(1):153. doi: 10.1186/s13643-016-0327-0 (PMC5016934; doi:10.1186/s13643-016-0327-0)
Supplement: Additional file 2: — Preliminary search strategy. Preliminary search strategy, including all queried databases, search parameters, and key words. (DOCX 112 kb) [file 13643_2016_327_MOESM2_ESM.docx]

**Additional file 2: Preliminary search strategy**

Database: Ovid MEDLINE(R) In-Process & Other Non-Indexed Citations and Ovid MEDLINE(R) <1946 to Present>, PsycINFO <1806 to March Week 2 2016>, Embase <1980 to 2016 Week 11>

Search Strategy:

--------------------------------------------------------------------------------

1 ((postoperati* or post-operati* or postsurg* or post-surg*) adj1 ("cognitive dysfunction" or "brain dysfunction")).tw. (1514)

2 Intensive Care Units/ (108148)

3 Burn Units/ (2664)

4 Coronary Care Units/ (11882)

5 Respiratory Care Units/ (105350)

6 exp Intensive Care Units, Pediatric/ (120476)

7 exp Critical Care/ (561981)

8 ((intensive or critical or acute) adj3 care).tw. (341890)

9 (ICU or ICUs or NICU or NICUs or PICU or PICUs or SICU or SICUs or CCU or CCUs).tw. (141438)

10 (burn$1 adj3 (unit$1 or centre$1 or center$1)).tw. (9497)

11 ((cardiac or coronary or heart) adj3 (unit$1 or centre$1 or center$1)).tw. (25672)

12 (respiratory adj3 (unit$1 or centre$1 or center$1)).tw. (7330)

13 ((surgical or surger*) adj3 (unit$1 or centre$1 or center$1)).tw. (39843)

14 Postoperative Care/ (126017)

15 Postoperative Complications/ (357273)

16 (postoperati* or post-operati* or postsurg* or post-surg*).tw. (1061551)

17 Critical Illness/ (43151)

18 (critical* adj (ill or illness*)).tw. (85986)

19 or/2-18 (2242314)

20 Delirium/ (27764)

21 deliri*.tw. (31886)

22 Psychoses, Substance-Induced/ (49999)

23 (psychos* adj3 (toxic* or exogenous* or chemical* or drug or drugs or medication* or substance*)).tw. (11249)

24 (acute brain adj (dysfunction* or failure* or syndrome*)).tw. (330)

25 (cloud* adj3 consciousness*).tw. (671)

26 clouded state*.tw. (14)

27 ((psycho-organic syndrome* or psychoorganic syndrome* or organic psychosyndrome* or organic psycho-syndrome*) adj3 acute).tw. (32)

28 exp Confusion/ci (1693)

29 Hallucinations/ (22742)

30 hallucinat*.tw. (40230)

31 or/20-30 (144204)

32 19 and 31 (13951)

33 1 or 32 (15205)

34 (controlled clinical trial or randomized controlled trial).pt. (494870)

35 clinical trials as topic.sh. (175313)

36 (randomi#ed or randomly or RCT$1 or placebo*).tw. (1777102)

37 ((singl* or doubl* or trebl* or tripl*) adj (mask* or blind* or dumm*)).tw. (340945)

38 trial.ti. (362588)

39 or/34-38 (2207460)

40 33 and 39 (1828)

41 exp Animals/ not (exp Animals/ and Humans/) (13482343)

42 40 not 41 (1198)

43 (comment or editorial or interview or letter or news).pt. (3067583)

44 42 not 43 (1187)

45 (2015* or 2016*).dc. (1335540)

46 44 and 45 (105) [MEDLINE RECORDS]

47 postoperative delirium/ (774)

48 postoperative cognitive dysfunction/ (501)

49 ((postoperati* or post-operati* or postsurg* or post-surg*) adj1 ("cognitive dysfunction" or "brain dysfunction")).tw. (1514)

50 intensive care psychosis/ (222)

51 or/47-50 (2588)

52 intensive care unit/ (144535)

53 burn unit/ (2803)

54 coronary care unit/ (11882)

55 intensive care/ (145804)

56 ((intensive or critical or acute) adj3 care).tw. (341890)

57 (ICU or ICUs or NICU or NICUs or PICU or PICUs or SICU or SICUs or CCU or CCUs).tw. (141438)

58 (burn$1 adj3 (unit$1 or centre$1 or center$1)).tw. (9497)

59 ((cardiac or coronary or heart) adj3 (unit$1 or centre$1 or center$1)).tw. (25672)

60 (respiratory adj3 (unit$1 or centre$1 or center$1)).tw. (7330)

61 ((surgical or surger*) adj3 (unit$1 or centre$1 or center$1)).tw. (39843)

62 postoperative care/ (126017)

63 postoperative complication/ (581063)

64 (postoperati* or post-operati* or postsurg* or post-surg*).tw. (1061551)

65 critical illness/ (43151)

66 (critical* adj (ill or illness*)).tw. (85986)

67 or/52-66 (2021179)

68 exp delirium/ (29751)

69 deliri*.tw. (31886)

70 (psychos* adj3 (toxic* or exogenous* or chemical* or drug or drugs or medication* or substance*)).tw. (11249)

71 (acute brain adj (dysfunction* or failure* or syndrome*)).tw. (330)

72 (cloud* adj3 consciousness*).tw. (671)

73 clouded state*.tw. (14)

74 ((psycho-organic syndrome* or psychoorganic syndrome* or organic psychosyndrome* or organic psycho-syndrome*) adj3 acute).tw. (32)

75 exp hallucination/ (44040)

76 hallucinat*.tw. (40230)

77 or/68-76 (109673)

78 67 and 77 (12740)

79 51 or 78 (14272)

80 randomized controlled trial/ or controlled clinical trial/ (1029410)

81 exp "clinical trial (topic)"/ (183678)

82 (randomi#ed or randomly or RCT$1 or placebo*).tw. (1777102)

83 ((singl* or doubl* or trebl* or tripl*) adj (mask* or blind* or dumm*)).tw. (340945)

84 trial.ti. (362588)

85 or/80-84 (2400800)

86 79 and 85 (2114)

87 exp animal experimentation/ or exp models animal/ or exp animal experiment/ or nonhuman/ or exp vertebrate/ (41561072)

88 exp humans/ or exp human experimentation/ or exp human experiment/ (32525665)

89 87 not 88 (9036994)

90 86 not 89 (2054)

91 (editorial or letter).pt. (2719941)

92 90 not 91 (2011)

93 (2015* or 2016*).dd. (2392153)

94 92 and 93 (304)

95 94 use emez (304) [EMBASE RECORDS]

96 ((postoperati* or post-operati* or postsurg* or post surg*) adj1 ("cognitive dysfunction" or "brain dysfunction")).tw. (1514)

97 exp intensive care/ (566304)

98 ((intensive or critical or acute) adj3 care).tw. (341890)

99 (ICU or ICUs or NICU or NICUs or PICU or PICUs or SICU or SICUs or CCU or CCUs).tw. (141438)

100 (burn$1 adj3 (unit$1 or centre$1 or center$1)).tw. (9497)

101 ((cardiac or coronary or heart) adj3 (unit$1 or centre$1 or center$1)).tw. (25672)

102 (respiratory adj3 (unit$1 or centre$1 or center$1)).tw. (7330)

103 ((surgical or surger*) adj3 (unit$1 or centre$1 or center$1)).tw. (39843)

104 postsurgical complications/ (736)

105 (postoperati* or post-operati* or postsurg* or post-surg*).tw. (1061551)

106 (critical* adj (ill or illness*)).tw. (85986)

107 or/97-106 (1911820)

108 delirium/ (27764)

109 deliri*.tw. (31886)

110 (psychos* adj3 (toxic* or exogenous* or chemical* or drug or drugs or medication* or substance*)).tw. (11249)

111 (acute brain adj (dysfunction* or failure* or syndrome*)).tw. (330)

112 (cloud* adj3 consciousness*).tw. (671)

113 clouded state*.tw. (14)

114 ((psycho-organic syndrome* or psychoorganic syndrome* or organic psychosyndrome* or organic psycho-syndrome*) adj3 acute).tw. (32)

115 mental confusion/ (188616)

116 exp Hallucinations/ (44040)

117 hallucinat*.tw. (40230)

118 or/108-117 (291591)

119 107 and 118 (16679)

120 96 or 119 (17930)

121 clinical trials/ (67543)

122 (randomi#ed or randomly or RCT$1 or placebo*).tw. (1777102)

123 ((singl* or doubl* or trebl* or tripl*) adj (mask* or blind* or dumm*)).tw. (340945)

124 trial.ti. (362588)

125 or/121-124 (1985120)

126 120 and 125 (2104)

127 exp Animals/ not (exp Animals/ and Humans/) (13482343)

128 126 not 127 (1295)

129 (2015* or 2016*).up. (28272758)

130 128 and 129 (519)

131 130 use prmz (499)

132 130 use emez (9)

133 130 not (131 or 132) (11) [PSYCINFO RECORDS]

134 46 or 95 or 133 (420) [ALL DATABASES]

135 remove duplicates from 134 (340) [TOTAL UNIQUE RECORDS]

136 135 use prmz (103) [UNIQUE MEDLINE RECORDS]

137 135 use emez (232) [UNIQUE EMBASE RECORDS]

138 135 not (136 or 137) (5) [UNIQUE PSYCINFO RECORDS]
